# A Case of Intracholecystic Papillary Neoplasm Extending Through a Cholecysto-Colonic Fistula

**DOI:** 10.7759/cureus.72854

**Published:** 2024-11-01

**Authors:** Shuichi Tsukamoto, Akihito Kozuki, Kunihiko Kaneda, Chihiro Ichikawa, Yukihiro Imai

**Affiliations:** 1 Department of Diagnostic Pathology, Kakogawa Central City Hospital, Kakogawa, JPN; 2 Division of Molecular and Genomic Pathology, Department of Pathology, Kobe University Graduate School of Medicine, Kobe, JPN; 3 Department of Surgery, Kakogawa Central City Hospital, Kakogawa, JPN

**Keywords:** cholecysto-enteric fistula, colon, gallbladder, intracholecystic papillary neoplasm, xanthogranulomatous cholecystitis

## Abstract

We report the case of a 76-year-old man who presented with pain in the right upper abdomen. Laboratory and radiological examinations revealed cholangitis, cholelithiasis, and a gallbladder tumor adhering to the transverse colon. After receiving conservative therapy for cholangitis, the patient underwent surgery for the gallbladder disease. On surgery, a cholecysto-colonic fistula communicating the gallbladder and transverse colon was revealed, and combined resection of the gallbladder and partial transverse colon was performed. The resected sample revealed an intracholecystic papillary neoplasm with a focal invasive component, xanthogranulomatous cholecystitis in the gallbladder, and a cholecysto-colonic fistula. Xanthogranulomatous cholecystitis caused fibrous adhesion and penetration from the gallbladder and transverse colon, resulting in the fistula. A noninvasive component of the intracholecystic papillary neoplasm horizontally extended into the transverse colon across the fistula, whereas a small invasive component on the hepatic side was observed. As the intracholecystic papillary neoplasm did not “invade” the transverse colon, we concluded that the pathological T-stage of the intracholecystic papillary neoplasm was pT1b (invading the muscularis propria of the gallbladder). This was a case where a large gap between preoperative diagnosis (cT3) and pathological diagnosis (pT1b) occurred and a careful explanation of the atypical state and cause of the discrepancy in diagnosis was required.

## Introduction

Intracholecystic papillary neoplasm (ICPN), a counterpart to pancreatic intraductal papillary mucinous neoplasm or intraductal papillary neoplasm of the bile duct, is a gallbladder tumor characterized by macroscopically well-defined and exophytic appearance and microscopically regular papillary structure. ICPN is basically a noninvasive tumor but may partially transform into an invasive carcinoma as it advances (ICPN with associated invasive carcinoma; ICPNAIC). ICPN is classified as low or high grade based on cytological or structural atypia; low-grade ICPN is regarded as a benign tumor (equivalent to an adenoma), whereas high-grade ICPN is regarded as a carcinoma in situ. The incidence of ICPN has been reported to be 0.4% of cholecystectomies. About half of ICPN patients document right upper abdominal pain and the other half have no symptoms. Therefore, a lot of ICPNs are found incidentally. Almost half of ICPNs are radiologically designated as gallbladder “cancer,” which indicates the difficulty in correct preoperative diagnosis of ICPN [1]. The current treatment strategy for ICPN is similar to that of conventional gallbladder carcinoma [2].

Various mimics of gallbladder carcinoma are known, and xanthogranulomatous cholecystitis (XGC) is one of them. XGC is a unique form of chronic cholecystitis in which bile leaks from the Rokitansky-Aschoff sinuses (RAS) and surrounding stroma, followed by a granulomatous reaction against bile acid and its metabolite, cholesterol [3]. This results in cholesterol deposits, inflammatory cell infiltration rich in foamy cells or giant cells, and fibrosis of the surrounding tissue. It frequently causes fibrous adhesions in the gallbladder and adjacent organs [3]. Mural thickening and the involvement of other organs can cause confusion in invasive gallbladder carcinoma, which sometimes leads to unnecessary surgery. However, XGC accompanies gallbladder carcinoma in 3%-12.5% of cases; thus, clinicians have difficulty in preoperative diagnosis or deciding on the therapeutic strategy [4,5].

Gallbladder diseases including acute or chronic cholecystitis, XGC, and gallbladder carcinoma sometimes affect the entire wall and extend to tissues or organs surrounding the gallbladder. These transmural lesions infrequently result in a fistula that connects the both gallbladder and gastrointestinal tract lumens. This fistula is referred to a cholecysto-enteric fistula (CEF). As CEF does not cause specific symptoms and is hard to be detected by imaging modalities, it is often diagnosed only during a surgery [6]. Although the pathogenesis of CEF has been reported or discussed, extension of a lesion across a CEF has rarely been discussed. Here, we report an extremely rare case of ICPN extending into the transverse colon via the CEF. This article was previously presented in a poster session at the 69th Autumn Annual Meeting of the Japanese Society of Pathology on November 9, 2023.

## Case presentation

**Figure 1:**
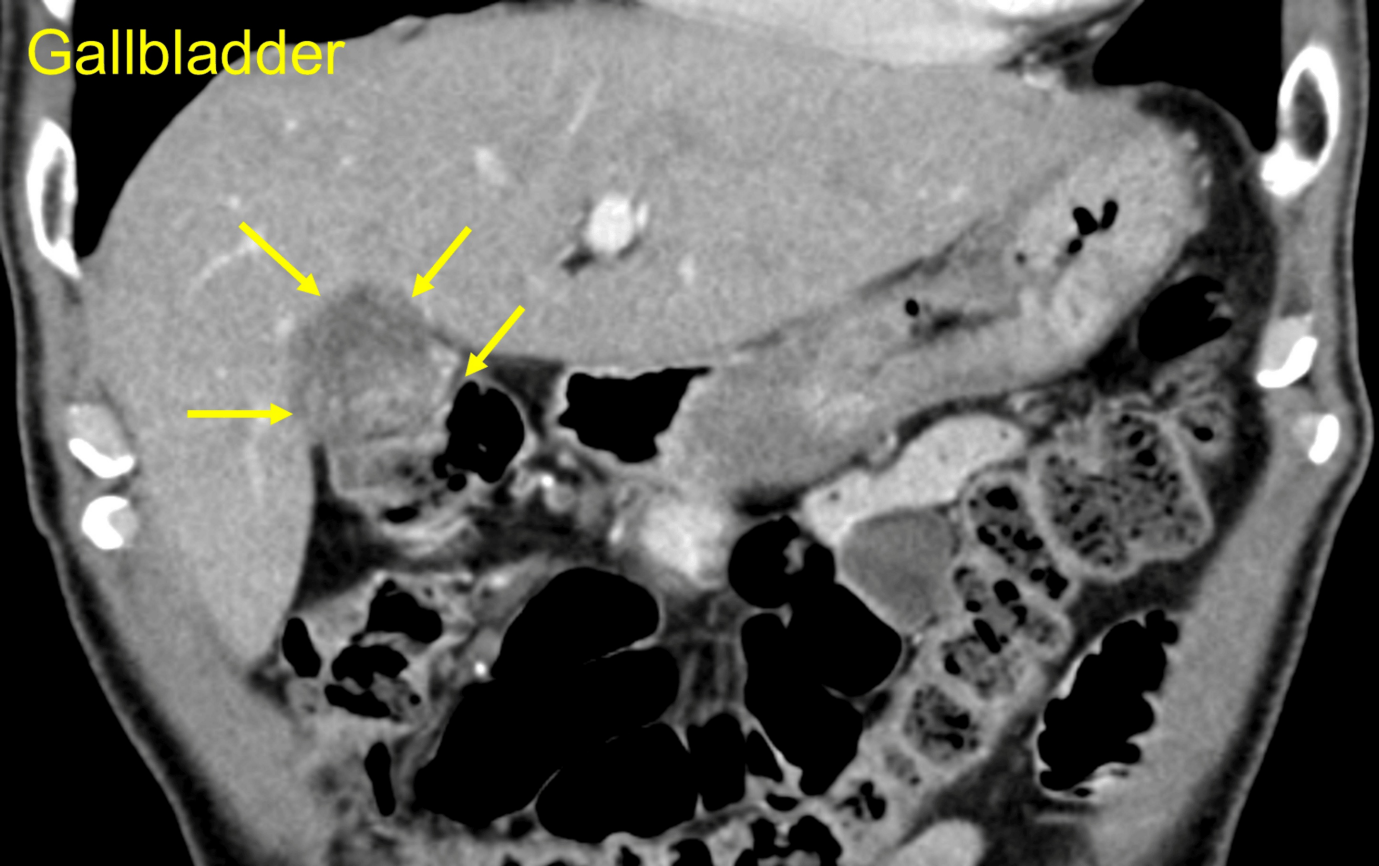
Abdominal computed tomography scan on admission Yellow arrows indicate the irregularly thickened gallbladder with a tumor filling the lumen. The border between the liver and gallbladder is unclear. The gallbladder seems to adhere to the transverse colon, suggesting gallbladder carcinoma invasion

A 76-year-old man presented to our hospital with right upper abdominal pain and a fever lasting for six days. The laboratory test results on admission are presented in Table 1. Briefly, the white blood cell counts and C-reactive protein, hepatobiliary enzyme, and bilirubin levels were elevated., suggesting an infectious disease caused by trouble in bile flow. Computed tomography (CT) scan demonstrated two calculi in the common bile duct. Tumors in the bile duct and pandcreatic head, or around the Vater’s ampulla were absent. Other specific findings that could cause the symptoms were not found. Collectively, he was diagnosed with cholangitis due to the calculi. The CT scan also revealed an irregularly thickened gallbladder adhering to the transverse colon (Figure 1), suggestive of gallbladder carcinoma with direct invasion to the transverse colon (clinical T3). He first underwent fasting and antibiotic therapy before the fever resolved on the next day of admission. After four days, endoscopic retrograde cholangiopancreatography was performed to remove the calculi. He was once discharged as his symptoms resolved, and about a month later, he was admitted again to receive surgery for the gallbladder disease. He underwent combined gallbladder and transverse colon resection.

**Table 1:** Laboratory tests on admission WBC: White blood cell; Hb: hemoglobin; PLT: platelet; CRP: C-reactive protein; AST: aspartate aminotransferase; ALT: alanine aminotransferase; γ-GTP: γ-glutamyl transpeptidase; ALP: alkaline phosphatase; IFCC: International Federation of Clinical Chemistry and Laboratory Medicine

Items	Value	Reference ranges
WBC	6600 /µL	4000-8000 /µL
Hb	10.4 g/dL	13.5-17.6 g/dL
PLT	152 x 10^3 ^/µL	158-348 x 10^3^ /µL
CRP	8.9 mg/dL	0.00-0.14 mg/dL
AST	140 U/L	13-30 U/L
ALT	234 U/L	10-42 U/L
γ-GTP	190 U/L	13-64 U/L
ALP (IFCC)	260 U/L	38-113 U/L
Total bilirubin	3.5 mg/dL	0.4-1.5 mg/dL
Direct bilirubin	1.8 mg/dL	0.0-0.4 mg/dL
Na	131 mEq/L	138-145 mEq/L
K	3.8 mEq/L	3.6-4.8 mEq/L
Cl	96 mEq/L	101-108 mEq/L

**Figure 2:**
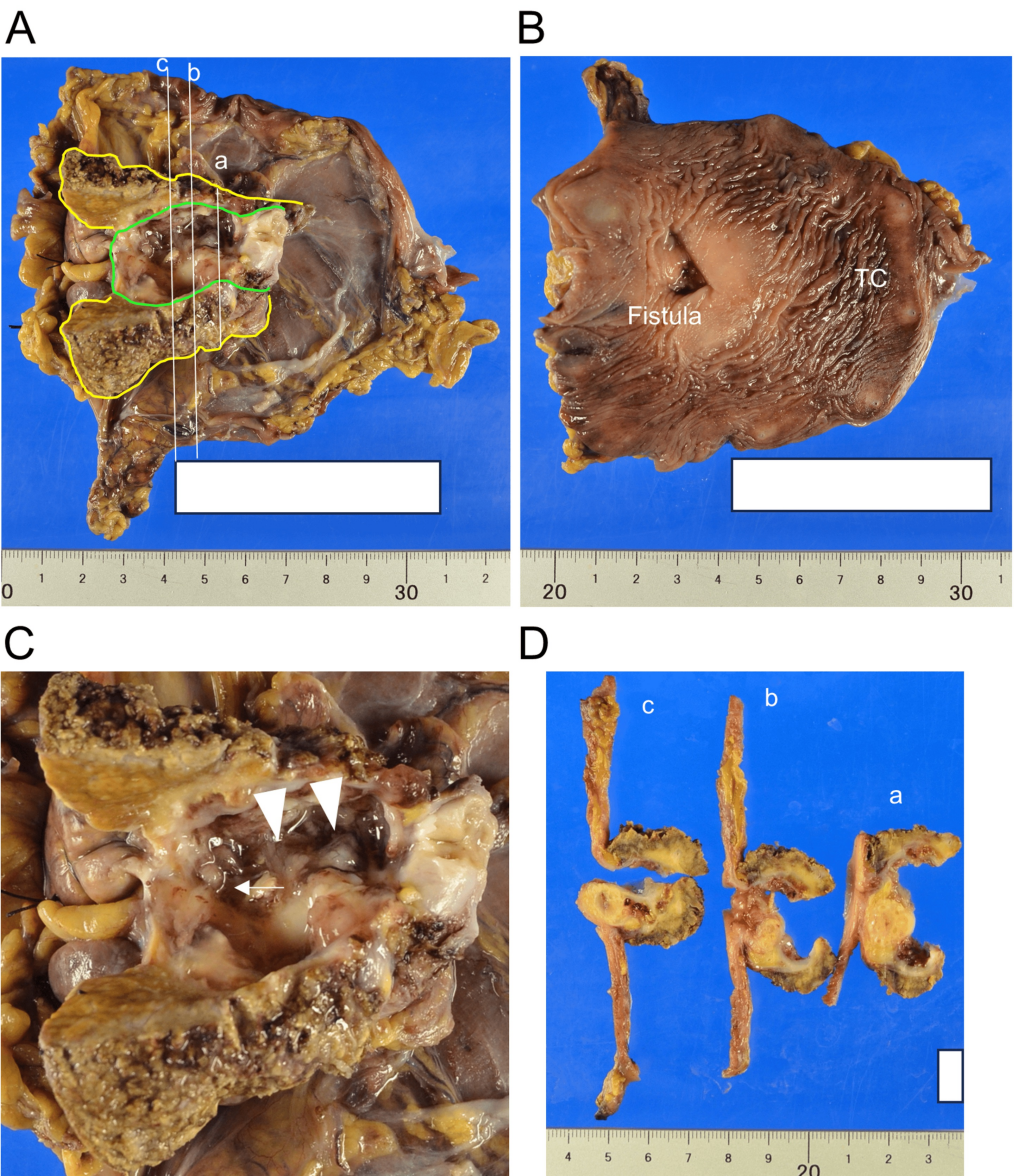
Macroscopic findings in surgically resected sample after formalin fixation TC: transverse colon (A) The gallbladder, gallbladder bed, and middle half of the transverse colon were resected. The mucosa of the incised gallbladder and serosal surface of the transverse colon are shown. The white lines represent the sectioning planes displayed in D, with planes b and c passing through the fistula. The yellow and green lines indicate the outlines of the liver and gallbladder, respectively. (B) Incised mucosal surface of the transverse colon with opening of the fistula. (C) Magnified view of the gallbladder lumen. Arrowheads indicate the papillary tumor. The arrow indicates the opening of the fistula in the gallbladder. (D) Cut surfaces of sliced samples, as shown in A. Fistulae appearing on planes b and c. The gallbladder wall was markedly thickened with scattered Rokitansky-Aschoff sinuses. Fibrosis involved the transverse colon and adjacent hepatic parenchyma

Macroscopically, the gallbladder wall exhibited diffuse thickening and was fibrously adhered to the transverse colon. Fibrosis affected the hepatic parenchyma of the gallbladder bed. Most of the gallbladder lumen was occupied by a papillary tumor (Figures 2A-2C). A small ulcer in the gallbladder body drained into a 16 mm-sized smooth, oval aperture on the colonic surface. It constituted a cholecysto-colonic fistula (Figures 2B-2D). Examination of the sliced surface revealed thickening of the gallbladder wall with fibrous muscular tissue and dilated RAS. Fibrosis affected the adjacent hepatic parenchyma and firmly adhered to the transverse colon. Notably, the colonic wall was retracted and torn, leading to the formation of a fistula between the gallbladder and the transverse colon (Figure 2D).

**Figure 3:**
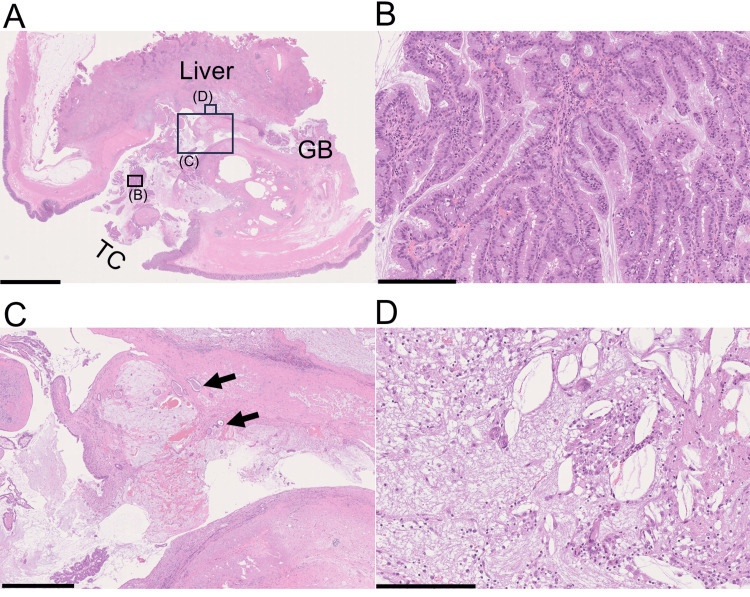
Slide-glass image of the cholecysto-colonic fistula and its neighboring structures GB: gallbladder; TC: transverse colon (A) The fistula connecting the lumens of both the gallbladder and transverse colon was evident. The gallbladder wall was thickened, with fibrous tissue and adenomyomatosis. Fibrosis involved the liver and the transverse colon. Frame rectangles (B), (C), and (D) are magnified in panel B, C, and D, respectively. (B) The papillotubular structure of the tumor was composed of columnar epithelium with foveolar-type features. (C) Arrows indicate tubular carcinoma components invading the fibrous or hyalinized gallbladder wall. (D) Abundant foamy macrophages, giant cells, and cholesterol clefts. Scale bar = 5 mm for A, 250 µm for B and C, 1 mm for D

**Figure 4:**
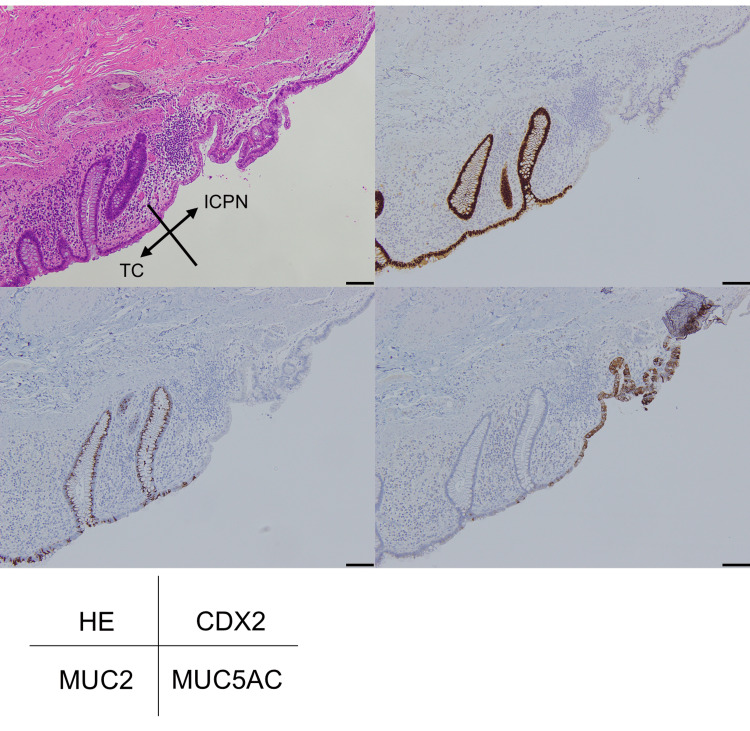
Border of the ICPN cell monolayer and the colonic epithelium on the colonic side of the fistula TC; transverse colon; ICPN; intracholecystic papillary neoplasm At the colonic end of the fistula, an epithelial cell transition from ICPN cells with a mucin cap resembling the gastric foveolar epithelium to colonic absorptive epithelial cells were observed (hematoxylin-eosin (HE), left top). ICPN cells are negative for CDX2 (right top) and MUC2 (left bottom), and positive for MUC5AC (right bottom). Scale bar = 100 µm

Microscopically, the gallbladder wall showed prominent fibrous thickening with scattered clusters of RAS and surrounding smooth muscle, indicating adenomyomatosis (Figure 3A). A xanthogranuloma comprising foamy macrophages, giant cells, and cholesterol clefts surrounded by a fibrous scar was observed between the gallbladder and the hepatic parenchyma of the gallbladder bed (Figure 3A). The gallbladder lumen was broadly occupied by foveolar-type ICPN with fernleaf-like papillary proliferation of tall columnar epithelium with rich apical mucin and basally located nuclei (Figure 3B). The gallbladder and transverse colon were adhered via the fibrous scar of the xanthogranuloma (Figures 3C-3D). This adhesion behaved as gallbladder carcinoma invasion on the preoperative CT scan. On the colonic side of the aperture, the monolayered ICPN epithelium extended through the fistula and confronted the colonic epithelium. Cancer invasion around the fistula was absent, indicating that ICPN extended into the colon only as a “horizontal extension.” Immunohistochemistry revealed a clear contrast between these two epithelial components: ICPN cells were positive for MUC5AC and negative for CDX2 and MUC2. Conversely, the colonic epithelium was positive for CDX2 and MUC2 and generally negative for MUC5AC (Figure 4).

A 1.5-2.0 mm-sized small invasive component of ICPN was observed on the gallbladder wall but not at the fistula or colon (Figures 3C, 4). We concluded that this tumor was an ICPNAIC of pT1b (invading the muscularis propria of the gallbladder) and not pT3 (invading the transverse colon). Surgical margin was negative for cancer, and the patient has been followed up with no additional treatment. He remains stable and no recurrence has been found at follow-up 19 months later from the surgery.

## Discussion

We encountered a case of ICPN extending into the transverse colon through a cholecysto-colonic fistula, and not by direct invasion. During the daily diagnostic process, it is often observed that ICPN cells replace the normal epithelium and extend far beyond macroscopic tumors. Preoperative overestimation of invasive gallbladder carcinoma is reported to be almost half that of ICPNs, as in the present case [1].

**Figure 5:**
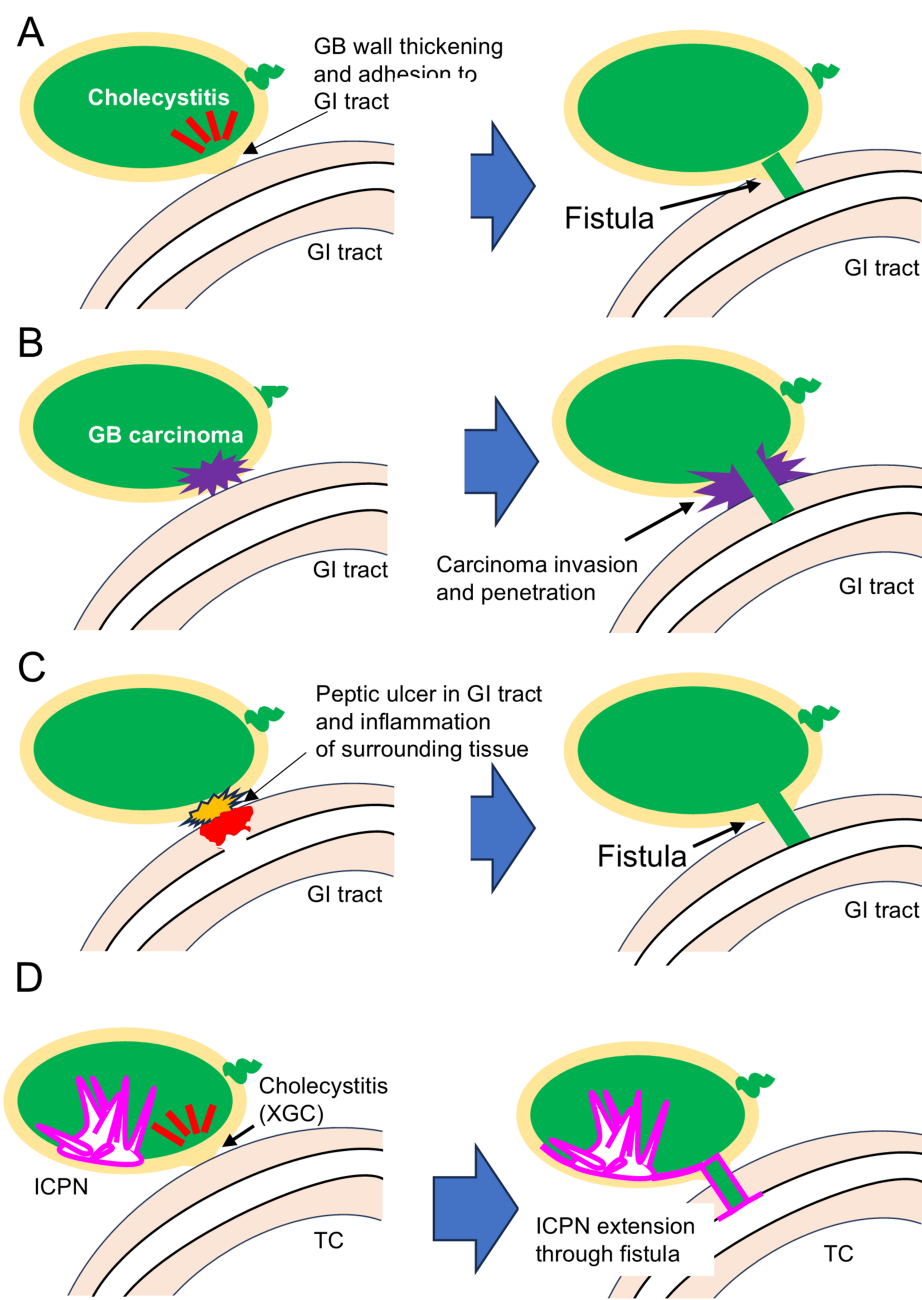
Schematic representation of cholecysto-enteric fistula formation GB: Gallbladder; GI: gastrointestinal; ICPN: intracholecystic papillary neoplasm; XGC: xanthogranulomatous cholecystitis; TC: transverse colon (A) Development of a cholecysto-enteric fistula secondary to chronic cholecystitis. Chronic cholecystitis results in fibrous adhesion to the gastrointestinal tract and subsequent fistula formation. (B) Development of a cholecysto-enteric fistula due to gallbladder carcinoma invasion. After a carcinoma invades the gallbladder serosa, it invades the gastrointestinal tract and forms a fistula. (C) Development of a cholecysto-enteric fistula caused by peptic ulcers. Gastroduodenal ulcers penetrating the gallbladder can result in fistula formation. (D) Schematic representation of the present pathology. A cholecystocolonic fistula consists of XGC through which ICPN cells extend horizontally into the transverse colon in a noninvasive manner. These images were created by the authors using Microsoft PowerPoint

CEF is a rare complication of various chronic cholecystic diseases (chronic cholecystitis, XGC, gallbladder carcinoma, etc.) and is infrequently acute (trauma, acute cholecystitis, etc.). CEF is categorized according to the organs communicating with the gallbladder: cholecysto-duodenal (60%-70%), -colonic (20%-30%), and -gastric (5%-10%) fistulas [3,7]. The common etiology of CEF is fibrous adhesion of the gallbladder and gastrointestinal tract walls, followed by destruction of both walls (Figure 5A). Cholecystitis, gallbladder carcinoma (Figure 5B), and peptic ulcers can also cause CEF (Figure 5C). Most cases of CEFs are observed during surgery because they do not cause specific preoperative symptoms [3]. In the present case, a cholecysto-colonic fistula was first observed during the surgery.

Collectively, pathologists should pay attention to the following points when evaluating malignancy with CEF: (1) the mechanism of formation of CEF and (2) the exact classification of pT stage for the malignancy. In this case, the surface of the fistula was lined with a monolayer of ICPN cells, whereas the walls of the gallbladder and transverse colon were tightly adhered by fibrosis across the fistula, containing the RAS, lymphocytes, and foamy cells. An invasive carcinoma component derived from the ICPN was absent from the fistula. These findings led us to conclude that the fistula was generated by XGC and extended to the transverse colon, but not by ICPNAIC. The next problem was evaluating the spread of ICPN, that is, pT classification. According to the latest Union for International Cancer Control TNM classification, ICPNAIC should be evaluated as in conventional gallbladder carcinomas. The present ICPN certainly “reached” the transverse colon, but whether this was pT3, which invades the transverse colon, was unclear. We believe it was not pT3, because the fibrous connection between the gallbladder and the transverse colon was due to XGC, not to a desmoplastic reaction associated with carcinoma invasion. A higher pT indicates a greater degree of vertical and destructive extension. Eventually, we determined the pT classification to be pT1b because a small gallbladder muscularis propria invasion was observed remotely from the fistula [8].

In conclusion, we encountered a rare gallbladder pathology in which ICPN extended horizontally into the transverse colon through the CEF (Figure 5D). This is a symbolic case with a large gap between the clinical (preoperative) and pathological diagnoses of cT3 and pT1b tumors. Considering the difficulty of preoperatively diagnosing CEF and discriminating between XGC and gallbladder carcinoma, this discrepancy may be inevitable. Therefore, careful gross and histological analysis is essential in such cases. Pathologists should carefully describe the atypical state and cause of the discrepancy in diagnosis to clinicians.

## Conclusions

We experienced an ICPN with unusual extension into the transverse colon through cholecysto-colonic fistula. As detecting CEF and correctly differentiating ICPN and gallbladder cancer were challenging, actual pathology (ICPN extending via CEF, pT1b) was far from the preoperative prediction (gallbladder carcinoma invasion to the transverse colon, cT3). Pathologists should keep in mind that unexpected disease spread sometimes occurs like in this case and should perform careful observation and grossing of surgical specimens. Cases like the present one are too rare to decide their stage easily because current cancer staging does not assume adjacent organ involvement "without cancer invasion." The present case may shed light on such a loophole in the present cancer staging.
